# Incidence of Circulating Antibodies Against Hemagglutinin of Influenza Viruses in Epidemic Season 2023/2024 in Poland

**DOI:** 10.3390/biom15070977

**Published:** 2025-07-07

**Authors:** Katarzyna Kondratiuk, Aleksander Masny, Anna Poznańska, Karol Szymański, Katarzyna Łuniewska, Emilia Czajkowska, Bartosz Mańkowski, Lidia B. Brydak

**Affiliations:** 1 Department of Virology, National Institute of Public Health NIH-National Research Institute, 00-791 Warsaw, Poland; 2 Department of Population Health Monitoring and Analysis, National Institute of Public Health NIH-National Research Institute, 00-791 Warsaw, Poland

**Keywords:** influenza, hemagglutinin antibodies, GMT, protection rate, serum, antibody, RNA viruses

## Abstract

The aim of this study was to determine the level of anti-hemagglutinin antibodies using the hemagglutination inhibition test (HAI) in the blood sera of patients collected during the 2023/2024 epidemic season in Poland. This data is valuable for assessing the level of population immunity to influenza viruses circulating in Poland during this epidemic season. The study material consisted of serum samples collected across the country and divided into seven age groups. The test results confirmed the presence of anti-hemagglutinin antibodies for the antigens included in the quadrivalent influenza vaccine recommended by the World Health Organization (WHO) for the 2023/2024 epidemic season: A/Victoria/4897/2022 (H1N1)pdm09, A/Darwin/9/2021 (H3N2), B/Austria/1359417/2021 (B/Victoria lineage) and B/Phuket/3073/2013 (B/Yamagata lineage). The highest values of the geometric mean (GMT = 121.0 [95% CI: 108.5–134.9]) and protective factor (70 [95% CI: 67–74]%) were recorded for the A/H3N2/influenza virus antigen. In Poland, the vaccination rate of the general population in the discussed season was only 5.52%. The obtained results can therefore be interpreted as a response of the immune system, consisting of the production of anti-hemagglutinin antibodies in patients who had previously had an infection caused by the influenza virus.

## 1. Introduction

Influenza is an acute infectious disease caused by the influenza virus, which occurs seasonally and manifests as an acute respiratory infection. What is characteristic of influenza is the sudden onset of its symptoms. Systemic symptoms often predominate over respiratory symptoms. The disease usually develops within 1–3 days after infection. A person infected with the influenza virus can transmit the virus 1–2 days before the onset of the first symptoms and remains contagious for the next 5–7 days. Children can spread the virus for up to 10 days. Experts point out that children are a reservoir of influenza viruses. They play a significant role in the transmission of the virus in society, including their parents, caregivers, the elderly, and other unvaccinated children. Therefore, vaccination of the youngest is of particular importance in protecting against this infection. In individuals with weakened immune systems, the contagious period may be even longer. The symptoms of influenza include fever or a subjective feeling of fever, chills, cough (usually dry), sore throat, runny or stuffy nose, muscle and joint pain, headache, fatigue and, in children, additional gastrointestinal symptoms, such as vomiting and diarrhea [1]. The World Health Organization (WHO) estimates approximately one billion seasonal influenza infections, 3–5 million severe cases of influenza, and up to 650,000 deaths due to post-influenza complications [2]. Influenza infection can lead to hospitalisation and severe complications, potentially resulting in respiratory failure and death. Influenza complications may emerge within the first few days of infection or even several weeks after the infection has resolved. It is estimated that 8.5% of hospitalisations for lower respiratory tract infections are caused by influenza viruses [3]. For these reasons, seasonal influenza represents a global public health problem and an economic burden on society [1,4,5].

There are three main types of influenza viruses: A, B, and C. Influenza viruses can be divided into subtypes based on the characteristics of their surface proteins, which are present on the virus surface: hemagglutinin (HA) and neuraminidase (NA). The specific combination of HA and NA not only defines the target cell and virulence of the influenza virus, but also affects its zoonotic potential and pandemic threat [4]. There are 11 subtypes defined by neuraminidase—NA (N1–N11) and 18 subtypes defined by hemagglutinin—HA (H1–H18). Influenza viruses are not classified into subtypes but are divided into two lineages: B/Victoria and B/Yamagata [6]. Type A influenza, which has pandemic potential, can occur in both humans and animals (horses, pigs, minks, and aquatic mammals such as seals and whales, as well as birds). All subtypes occur in birds but only a few are found in humans (H1–H3; N1, N2), pigs (H1N1), and horses (H3N8; H7N7). The new pandemic strains of influenza A virus arise by the reassortment of genes from human and animal strains during a dual infection in an intermediate host, probably pigs, which constitute the so-called “mixing vessel” [7]. Type B influenza virus infects only humans and the disease it causes is usually milder compared to infection with type A influenza. Type B influenza is most often endemic, and outbreaks of the virus typically occur at intervals of 2–3 years [8].

The most characteristic feature of the influenza virus is its antigenic variability. Influenza viruses undergo frequent antigenic changes, resulting from point mutations occurring during replication, which lead to the emergence of new virus variants. This distinguishes influenza from other viruses, thus posing a significant public health threat.

The most important membrane protein of the influenza virus is hemagglutinin (HA). This multifunctional protein is composed of three polypeptide chains consisting of 550 amino acids. HA is located on the surface of the influenza virus and is responsible for its entry into the host cell. It facilitates the adsorption of the virus to the cellular receptor. HA also possesses membrane fusion capabilities, enabling the viral envelope to merge with host cell membranes, allowing the virion to enter the cytoplasm and release its internal components [4,9]. The induction of neutralising antibodies against hemagglutinin plays a key role in generating protection against influenza in humans [8]. Influenza viruses undergo genetic changes via antigenic drift and antigenic shift. Antigenic drift refers to genetic variability caused by point, spontaneous mutations occurring during the replication of influenza viruses. Mutation is a random phenomenon and its frequency depends, among other factors, on the efficiency of the nucleic acid replication and repair apparatus [9]. In influenza B viruses, antigenic drift occurs significantly slower than in influenza A viruses. Each mutation that facilitates immune evasion in the infected host undergoes positive selection, is passed on to subsequent generations and spreads more widely. A similar phenomenon of antigenic variability in influenza viruses is antigenic shift, which involves the reassortment of one or more segments of the single-stranded RNA of the influenza virus. Antigenic shift results in the emergence of a new subtype of influenza A virus capable of causing a pandemic. Antigenic changes occur within the surface antigens of the influenza virus—hemagglutinin and neuraminidase [10].

The most important element of the influenza control strategy is the seasonal use of vaccines, which are a scientifically proven method for preventing and combating seasonal influenza epidemics in infected populations. In Poland, inactivated vaccines are used for influenza prevention, especially the so-called “split” vaccines (with a split virion), “subunit” (subunit, containing purified surface antigens: neuraminidase and hemagglutinin), and “live” intranasal vaccines (with an attenuated virion) [11].

The emergence of new influenza virus mutations through antigenic drift can explain seasonal influenza epidemics worldwide, necessitating annual changes to the composition of the influenza vaccine.

The aim of the study was to determine the average level of anti-hemagglutinin antibodies and protective titres against influenza viruses in the Polish population during the 2023/2024 influenza epidemic season. The study primarily focuses on anti-hemagglutinin antibodies, which are produced during an influenza infection or after vaccination against influenza. These antibodies are responsible for blocking the ability of the influenza virus to adsorb to host cells. They mainly belong to the IgG class. The severity of clinical symptoms is inversely proportional to the level of antibodies in the serum for hemagglutinin. At high concentrations, antibodies to hemagglutinin provide complete protection against influenza infection, while at lower concentrations, they alleviate disease symptoms. Unfortunately, antibodies targeting one type or subtype of influenza virus do not provide protection against infection by a different type or subtype of the virus [1,4]. Moreover, even within the same subtype, antibodies targeting one strain do not always protect against infection by another strain that differs antigenically [12,13]. A study was conducted using a serological hemagglutination inhibition test (HAI), which involves detecting antibodies that specifically inhibit hemagglutination reaction, following a previous influenza infection or influenza vaccination. This test is used to diagnose viruses that posses hemagglutinin, capable of agglutinating (clumping) erythrocytes, including influenza viruses. In this test, the viral antigen is added to the patient’s diluted serum. The anti-hemagglutinin antibodies in the serum bind to the specific hemagglutinin, blocking its activity. After the addition of red blood cells, no clumping is observed. This means that the blood will clot upon the addition of these viruses, as the red blood cells bind to the hemagglutinin on the virus surface. If the serum sample contains specific antibodies against hemagglutinin, the addition of the influenza virus causes an antigen/antibody reaction. This is visible after the next addition of red blood cells, which are no longer agglutinated because the reaction is inhibited. The reciprocal of the highest dilution at which the inhibition of agglutination is still observed determines the antibody titre. HAI is a test used to measure humoral immunity developed after influenza infection [14].

The National Influenza Center (NIC) in Poland takes an active part in the prevention, control, and monitoring of influenza. The monitoring of influenza involves activities aimed at tracking the spread of the influenza virus and assessing its activity. This allows us to predict the future waves of the disease, update the vaccines and adapt the prevention and treatment strategies. The NICs are national institutions designated by the ministries of health and recognized by the WHO. The NIC in Poland is the backbone of the WHO’s Global Influenza Surveillance and Response System (GISRS). The National Influenza Center (NIC) takes an active part in the activities of the network for the prevention and control of influenza. The tasks of the NIC also include assessing the level of immunity of the population to the strains of influenza virus circulating in Poland in any given season. This assessment is based on serological tests, i.e., using the hemagglutination inhibition assay (HAI). The study results provide a serological overview of the course of the influenza epidemic season. This is also important from the perspective of influenza surveillance in Poland, as not every patient with flu-like symptoms undergoes PCR testing to confirm influenza virus infection. Determining antibody levels and establishing whether a given patient had a protective titre provides information about prior exposure to the virus [15,16].

## 2. Materials and Methods

A total of 700 blood serum samples collected from patients were tested. The samples were collected by 16 Provincial Sanitary and Epidemiological Stations in Poland, following the recommendations of the WHO [17]. Samples were collected during the 2023/2024 influenza epidemic season, i.e., between 1 October 2023 and 30 September 2024. The number of tested serum samples from each voivodeship varied. The sera were categorised into 7 age groups based on patient age, with 100 individuals in each of the following groups: 0–4 years of age, 5–9 years of age, 10–14 years of age, 15–25 years of age, 26–44 years of age, 45–64 years of age, and ≥65 years of age. Prior to testing, the samples were stored frozen at −80 °C.

The study used influenza virus strains included in the 2023/2024 seasonal influenza vaccine for the Northern Hemisphere, including Poland, as recommended by the WHO.

The viruses were obtained from the Worldwide Influenza Centre (NIC) at the Crick Institute, London, thanks to close collaboration within the WHO Global Influenza Surveillance Network. They were propagated to the quantity required for the study at the National Influenza Center of the National Institute of Public Health NIH—National Research Institute (NIZP PZH-PIB), following WHO recommendations [17], using propagation in the allantoic cavity of 11-day-old embryonated chicken eggs. After inoculation with the appropriate virus, the embryos were incubated under the following conditions: for A/H3N2/and A/H1N1/pdm09 viruses (2 days at 37 °C) and for influenza B viruses (3 days at 35 °C). After incubation, the titres of the propagated viruses were determined. Prior to use in the study, appropriately labelled virus vials were stored at −80 °C. Influenza virus strains used for the hemagglutination inhibition assay (HAI) in the 2023/2024 epidemic season recommended by WHO are presented in Table 1.

In the study, the level of anti-hemagglutinin antibodies, specific antibodies against influenza, was measured using the hemagglutination inhibition assay (HAI). The sera used in the study were inactivated according to the recommendations [17,18]. The viruses were prepared to a titre of 1:8 hemagglutination units. Transparent microtitre plates with a V-shaped bottom were used for the test. The necessary turkey red blood cells (RBC) for the test were suspended in Alsever’s solution, which acts as an anticoagulant and blood preservative, and were delivered to the laboratory in this form. The turkey red blood cell concentrate was obtained by centrifugation at 1200 rpm for 10 min. In this study, red blood cells with an RBC concentration of 0.5% were used, obtained by using a ratio of 999 µL PBS and 5 µL packed blood cells. Immediately before the test, each serum sample was treated with receptor-destroying enzyme (RDE) (Thermo Fisher Scientific) at 37 °C for 16 h. After this, the enzyme in the serum–RDE mixture was inactivated by incubation at 56 °C for 30 min. The prepared sera were then used in the HAI test, where serial dilutions were made in PBS buffer. The serum samples were diluted in 96-well polypropylene plates with a U-shaped bottom; 50 µL of serum was pipetted into the first row of the plate, followed by serial dilutions with 25 µL of PBS. To these diluted sera, 25 µL of virus at a titre of 1:8 was added, and the plates were incubated at room temperature for 15 min. After this period, 50 µL of blood cell solution was added to all wells and the plates were incubated at room temperature for 30 min before the results were read. In the event of an antigen–antibody reaction, the hemagglutination of erythrocytes on the plate is inhibited. The occurrence of hemagglutination indicates that the antigen is homologous to the specific antibody added. For each tested sample, the highest serum dilution that inhibits hemagglutination must be recorded. The titres of anti-hemagglutinin antibodies are as follows: 10, 20, 40, 80, 160, 320, etc. [17,19].

The obtained study results were analysed by calculating the following parameters: the prevalence of specific anti-hemagglutinin antibodies in the serum samples from the studied age groups, the geometric mean antibody titres (GMT), and the protective rate (%) (i.e., the percentage of individuals with anti-hemagglutinin antibodies at a level of ≥1:40, which was obtained either after vaccination against influenza or after a previous infection caused by the influenza virus).

## 3. Statistical Analysis

All of the analysed values were determined along with the 95% confidence interval limits. The chi-square test was used to compare age groups regarding the categorical variables (prevalence of anti-hemagglutinin antibodies and reaching their protective titre). The Kruskal–Wallis test was applied to compare the titre distribution between seven age groups, and the Mann–Whitney U test was used for two (children up to 14 years of age vs. the older population aged 15+). For all the tests, the significance level was 0.05. The calculations were executed with the Stata/IC 16 software.

## 4. Results

**Figure 1:**
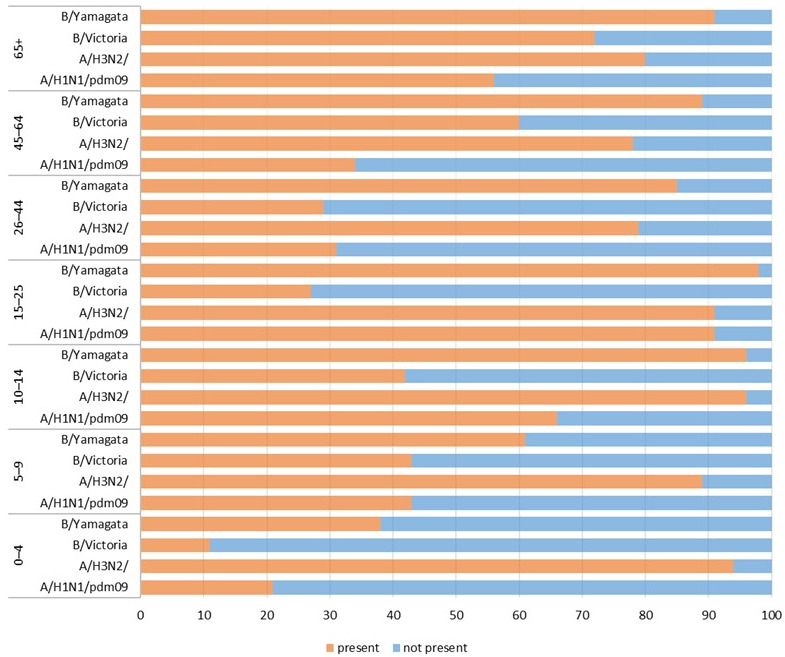
The presence of antibodies in the serum of patients aged 0–4, 5–9, 10–14, 15–25, 26–44, 45–64, and 65+ years of age in the 2023/2024 epidemic season.

The results of the study confirmed the presence of antibodies against A/Victoria/4897/2022 (H1N1)pdm09 in 342 patients (49 [45–53]% of all 700 tested individuals, with the 95% confidence interval in square brackets). The largest number of patients tested had antibodies against A/Darwin/9/2021 (H3N2) in their sera—607 patients (87 [84–89]% of the tested samples). Antibodies against B/Austria/1359417/2021 (B/Victoria lineage) were detected in the smallest number of samples—284 patients (41 [37–44]% of those tested); 558 patients had antibodies against B/Phuket/3073/2013 (B/Yamagata lineage) (80 [77–83]% of the samples tested). Figure 1 shows the percentage of patients in individual age groups (children and adults) that had antibodies against a particular influenza virus in the 2023/2024 epidemic season.

The prevalence of all antibodies analysed in the study differed significantly (for all antibodies, *p* < 0.001). The prevalence of antibodies against A/Victoria/4897/2022 (H1N1)pdm09 ranged from 21 [13–29]% in the 0–4 age group to 91 [85–97]% in the 15–25 age group. The prevalence of antibodies against A/Darwin/9/2021 (H3N2) ranged from 78 [70–86]% in patients aged 45–64 years to 96 [92–100]% in those aged 10–14 years. The prevalence of antibodies against B/Austria/1359417/2021 ranged from 11 [5–17]% in the 0–4 age group to 72 [63–81]% in the oldest patients over 65 years of age. The prevalence of antibodies against B/Phuket/3073/2013 ranged from 38 [28–48]% in the 0–4 age group to 98 [95–100]% in the 15–25 age group.

The geometric mean titres (GMT) for individual antigens are presented in Table 2. The highest GMT value among all the influenza viruses tested was observed for the hemagglutinin A/H3N2/(21.0 [108.5–134.9]), while the lowest was for B/Austria/1359417/2021 (B/Victoria lineage)—34.9 [30.9–39.4].

**Figure 2:**
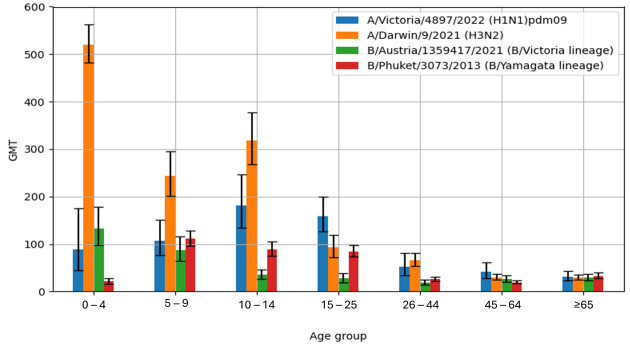
Geometric mean with 95% confidence intervals for titres of anti-hemagglutinin antibodies in patient sera according to age in the 2023/2024 epidemic season in Poland.

Figure 2 shows the geometric mean titres of anti-hemagglutinin antibodies (GMT) in the sera of patients in the particular age groups in the 2023/2024 epidemic season in Poland.

The highest geometric mean antibody titre (GMT) for A/H1N1/pdm09 hemagglutinin was found in the sera of patients aged 10–14 years (GMT = 181.5 [133.6–246.5]), while the lowest GMT value was observed in the oldest age group, ≥65 years (GMT = 31.6 [23.5–42.5]). The GMT values for haemagglutinin A/H3N2/were the highest in the youngest patient group, 0–4 years of age (GMT = 520.6 [481.8–562.5]). The lowest value for the A/H3N2/influenza virus was found in the sera from patients aged 45–64 years (GMT = 29.8 [24.4–36.4]). The GMT value for the influenza B antigen B/Austria/1359417/2021 (B/Victoria lineage) reached the highest value in the sera of the youngest children aged 0–4 years (GMT = 132.4 [98.0–179.0]), while the lowest GMT value was confirmed in the sera from patients in the 26–44 years age group (GMT = 19.1 [14.4–25.3]). The highest GMT value for influenza B/Phuket/3073/2013 (B/Yamagata lineage) antigens was found in the sera of children aged 5–9 years (GMT = 111.2 [96.0–128.9]), while the lowest GMT value was observed in patients aged 45–64 years (GMT = 19.5 [16.6–23.0]).

The obtained study results also allowed the calculation of the protection rate (%), defined as the percentage of individuals with anti-hemagglutinin antibodies titres of ≥1:40.

**Figure 3:**
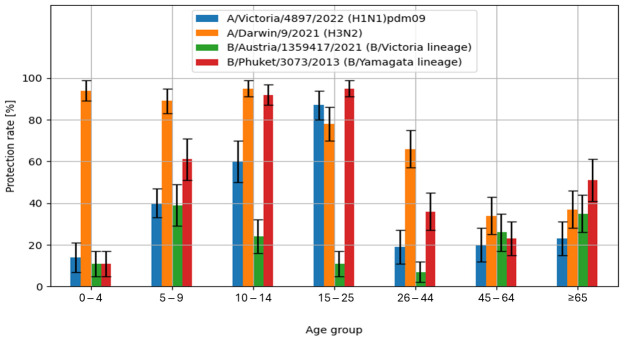
Percentage of cases with protective anti-hemagglutinin antibody titre (%), i.e., ≥40, with 95% confidence intervals in the 2023/2024 epidemic season, by age.

The highest values of the protection rate were recorded for the A/H3N2/influenza virus antigen in the sera of patients aged 10–14 years (95 [91–99]%) and in the youngest patients aged 0–4 years (94 [89–99]%). Similar values were observed for the B/Phuket/3073/2013 influenza antigens in serum samples from patients aged 15–25 years (95 [91–99]% and 10–14 years (92 [87–97]%). The lowest protection rate was found in the tested patients aged 26–44 years (7 [2–12]%) for the B/Austria/1359417/2021 antigen. Figure 3 shows the percentage of cases with protective anti-hemagglutinin antibody titre (%), i.e., ≥40, in the 2023/2024 epidemic season, in various age groups.

Analysis of the study results shows that individuals aged 15 years or older had a significantly higher frequency of antibodies against influenza A/Victoria/4897/2022 (H1N1)pdm09 compared to children under 15 years of age (53 [43–63]% vs. 43 [34–53]% (*p* = 0.011), as well as antibodies against influenza B/Austria/1359417/2021 (47 [37–57]% vs. 32 [23–41]% (*p* < 0.001) and B/Phuket/3073/2013 91 [85–96]% vs. 65 [56–74]% (*p* < 0.001).

Children under 15 years of age had a significantly higher frequency of antibodies against A/Darwin/9/2021 (H3N2) compared to older individuals (93 [88–98]% vs. 82 [74–90]%, *p* < 0.001). Determination of the prevalence of antibodies, GMT values, and the protective level total was carried out in two groups—children up to 14 years of age and the older population (aged 15+)—as shown in Table 2.

## 5. Discussion

The analysis of the study results confirms the circulation in the Polish population of all four influenza virus strains included in the composition of the quadrivalent influenza vaccine for the 2023–2024 epidemic season in the Northern Hemisphere, including Poland: A/Victoria/4897/2022 (H1N1)pdm09-like virus, A/Darwin/9/2021 (H3N2)-like virus, B/Austria/1359417/2021 (B/Victoria lineage)-like virus, and B/Phuket/3073/2013 (B/Yamagata lineage)-like virus. The vaccine composition was recommended by the World Health Organisation (WHO) for the 2023/2024 influenza epidemic season in the Northern Hemisphere.

Based on the obtained results, it can be observed that for all influenza antigens considered, the GMT value was significantly and statistically higher (*p* < 0.001) in children aged 0–14 years. The GMT values for the influenza antigens A/H1N1/pdm09, A/H3N2/, B/Austria/1359417/2021, and B/Phuket/3073/2013 for children aged 0–14 years were 135.6 [108.7–169.3], 344.8 [313.5–379.2], 61.2 [49.9–75.2], and 72.2 [62.9 –82.8], respectively. For comparison, the GMT values for the above influenza virus antigens in adult patients aged 15 years and older were as follows: 71.1 [59.5–85.0], 49.6 [44.1–55.8], 26.2 [22.9–30.0], and 35.4 [32.0–39.2]. A similar trend was observed during the previous 2022/2023 epidemic season, but the difference was statistically significant only for the B/Phuket/3073/2013 antigen [20].

In only the case of influenza A/H3N2/, the examined children aged 0–14 years achieved a protective level of anti-hemagglutinin antibodies of ≥1:40 significantly more likely than adults aged 15 and over (93 [88–98]% vs. 54 [44–64]%, *p* < 0.001). For A/H1N1/pdm09 and influenza B viruses, the differences were negligible (for A/H1N1/pdm09 in children 38 [28–48]% and 37 [28–47]% in adults, for B/Austria/1359417/2021 in children 25 [16–33]% and in adults 20 [12–28]%, for B/Phuket/3073/2013–55 [45–64]% in children and 51 [41–61]% in adults).

The highest number of tested patients had antibodies against A/Darwin/9/2021 (H3N2) in their serum. A similar situation occurred in previous influenza seasons—2021/2022 and 2022/2023 [20,21].

Antibodies against influenza virus haemagglutinin (HA) are considered the main correlate or mediator of protection against influenza virus infection. These antibodies are produced in the body following influenza vaccination or as a result of infection with the influenza virus. HA-specific antibodies mainly bind to the head domain of HA and block its interaction with the sialic acid receptors of the host cell. It is assumed, based largely upon population and aggregate data, that high titres (≥1:40) of anti-haemagglutinin antibodies provide good protection against influenza infection [22,23]. The U.S. Food and Drug Administration and the Committee for Medicinal Products for Human Use of the European Medicines Agency also define a HAI titre ≥1:40 as the main correlate of protection against influenza infection [15]. For inactivated influenza vaccines containing viral HA, a HI titre of 1:40 has been suggested as a reasonable statistical correlate for 50–70% efficacy in preventing the clinical symptoms of influenza, based on challenge studies in healthy adults.

Neuraminidase-inhibiting antibodies have also been shown to contribute to immunity. Although neuraminidase antibodies, even at high titres, do not prevent influenza infection, they can inhibit replication, thereby attenuating the course of infection caused by influenza virus infection [22,23,24].

Monitoring the circulating influenza virus strains in the population is essential for planning future vaccination strategies to ensure protection against influenza. High vaccination rates reduce the spread of the influenza virus. It also reduces the risk of a seasonal epidemic. Investing in the influenza vaccine is economically beneficial, as it reduces the burden on the healthcare system. It protects not only vaccinated people, but also those who cannot be vaccinated (e.g., people with medical contraindications). High vaccination rates improve the country’s health indicators (e.g., life expectancy and the quality of health care). The World Health Organization (WHO) emphasizes that influenza vaccination is a highly cost-effective public health measure. According to the WHO’s economic guidance, the cost of vaccinating individuals—especially those in high-risk groups like older adults or people with chronic conditions—is significantly lower than the direct medical costs of treating influenza-related complications, including hospitalizations [25]. High vaccination rates reduce the risk of concurrent occurrences of influenza, COVID-19, or other viral infections, which could significantly overwhelm the healthcare system.

Because protection against influenza after vaccination lasts approximately 6–12 months, the composition of vaccines recommended by the WHO changes each influenza season, based on circulating strains, as the continuous genetic evolution of the virus leads to antigenic variability. These strains are selected based on predictions from influenza virus surveillance systems. Seasonal vaccinations are the most effective way to prevent the infections and severe complications caused by influenza, as recommended by the World Health Organization and epidemiological experts [23,26,27,28]. They are the most cost-effective strategy for preventing influenza [29]. Seasonal influenza vaccination has been proven to be safe and well-tolerated by individuals of all ages [30]. The influenza vaccination is especially important for people at high risk of severe influenza, including those at increased risk of hospitalization and death. These include, for example, individuals over 65 years of age. Adults aged 65 and older are at greater risk of serious influenza complications due to both the increased likelihood of chronic diseases and a weakening of the immune system with age. Centres for Disease Control and Prevention (CDC) estimates that, in recent years, 70% to 85% of seasonal influenza-related deaths occurred among people aged 65 and over and 50% to 70% of influenza-related hospitalisations occurred in this age group [31]. The WHO and ECDC recommend achieving a vaccination rate of 75% among the senior population to achieve a positive effect of vaccination in the population [32]. As of 1 October 2024, a high-dose influenza vaccine has been available in Poland for individuals aged 60 and older. It has been developed for the elderly, for whom standard vaccines may not induce a sufficient immune response. The four-fold higher antigen dose (60 mcg vs. 15 mcg) in the high-dose vaccine results in a stronger immune response, and therefore, higher efficacy and better protection against the influenza virus in this age group. Studies have shown that the high-dose vaccine provides 24.2%-better protection against laboratory-confirmed influenza and reduces the risk of influenza-related hospitalisations by 11.2%. This vaccine is also more effective in preventing severe pneumonia (39.8%), cardiorespiratory complications (17.7%), hospitalisations due to respiratory diseases (14.7%), and hospitalisations for cardiovascular reasons (12.8%). A meta-analysis covering 12 influenza seasons confirms that the high-dose vaccine significantly reduces the risk of infection, hospitalisation, and complications [33,34,35].

Seasonal influenza vaccines achieve 70–90% protection rates when the vaccine components closely match circulating strains, and the vaccinated individuals are of normal immunity. Smaller benefits are usually observed in patients with weakened immune systems [10]. In the adjusted analysis, the estimated effectiveness of the 2023/2024 influenza vaccine against medically necessary illness due to influenza A and B was 41%. Stratified by influenza type and subtype, the estimated effectiveness against A(H1N1)pdm09, B/Victoria, and A(H3N2) was 28%, 68%, and 30%, respectively [36].

In Poland, there has been a long-standing low acceptance of influenza vaccinations, as evidenced by data on vaccination coverage across the entire population. In terms of vaccination rates, we have among the lowest in Europe. In the 2023/2024 epidemic season, despite the introduction of numerous positive systemic changes, including broad funding for influenza vaccines and the involvement of pharmacists in administering adult vaccinations, we are deeply concerned to observe another year of decline in the number of people receiving influenza vaccination. The vaccination rate for the general population in Poland in the 2023/2024 season was 5.52% (compared to 5.65% in the 2022/2023 season and 6.90% in the 2021/2022 season). Most likely due to the introduction of free influenza vaccines in Poland in the 2023/2024 epidemic season, vaccination rates increased in the 0–4 population: 123% increase to 4.7% (2.1% in the 2022/2023 season), children aged 5–14: 47% increase to 2.8% (1.9% in the 2022/2023 season), and in the 65+ population: 2% increase to 20.5% (20.1% in the 2022/2023 season). In the 15–64 age group, the vaccination rate in Poland in the 2023/2024 season was 1.8% [37]. By comparison, Denmark and Ireland have flu vaccination rates above 75% among people aged 65 and over, indicating a significant difference in the availability and acceptance of the vaccinations in these countries [38]. Given the low vaccination rates, these results underscore the need for increased efforts from health authorities to promote vaccination and provide clear recommendations to counter misinformation and ensure accurate information on vaccine safety. To increase the level of vaccination against influenza in Poland, social education at schools and informational campaigns at workplaces could be useful. In order to increase their influence on society, the participation of well-known and trusted people, as well as supporting doctors and pharmacists, is recommended.

## 6. Conclusions

Analysis of hemagglutination-inhibiting antibody levels in sera collected from patients across age groups during the 2023/2024 influenza season led to the following conclusions:

- Our studies have confirmed in the sera of patients of different ages that the presence of antihemagglutinin antibodies against four strains of influenza virus included in the quadrivalent influenza vaccine for the 2023/2024 epidemic season in the Northern Hemisphere, including Poland, was confirmed in the Polish population: the A/Victoria/4897/2022 (H1N1)pdm09-like virus, A/Darwin/9/2021 (H3N2)-like virus, B/Austria/1359417/2021 (B/Victoria lineage)-like virus, and B/Phuket/3073/2013 (B/Yamagata lineage)-like virus. The vaccine composition was recommended by the World Health Organisation (WHO) for the 2023/2024 influenza season in the Northern Hemisphere.
- The highest values of the geometric mean titre (GMT) and protective rate (%) were recorded for the A/H3N2 influenza virus antigen.
- In the 2023/2024 influenza season, the vaccination coverage rate remained low, as in previous seasons. For this reason, the observed antibody titres and protective rates may have resulted from immune responses and the development of anti-hemagglutinin antibodies in patients who had previously been infected with the influenza virus.1.

This data emphasizes the need to increase the level of vaccination against influenza in Poland, especially among the elderly. The introduction of effective educational programs, facilitating access to vaccinations, and informational campaigns can contribute to improve these indicators.

## Figures and Tables

**Table 1:** Influenza virus strains used for the hemagglutination inhibition assay (HAI) in the 2023/2024 epidemic season.

Antigens for the Epidemic Season 2023/2024		
Influenza virus strains	A/H1N1/pdm09	A/Victoria/4897/2022 (H1N1)pdm09-like virus
	A/H3N2/	A/Darwin/9/2021 (H3N2)-like virus
	B Victoria lineage	B/Austria/1359417/2021 (B/Victoria lineage)-like virus
	B Yamagata lineage	B/Phuket/3073/2013 (B/Yamagata lineage)-like virus

**Table 2:** Determination of the prevalence of antibodies, GMT values, and the protective level total in two groups—children up to 14 years of age and the older population (aged 15+).

Parameter	Total	Children 0–14 years	15+ years	Statistical Significance of Difference
Number of subjects	700	300	400	
	**A/Victoria/4897/2022 (H1N1)pdm09**			
% with antibodies	49% [45–53%]	53 [[43–63]%	43 [34–53]%	*p* = 0.011
GMT	90.9 [78.8–104.8]	135.6 [108.7–169.3]	71.1 [59.5–85.0]	*p* < 0.001
Protection rate	38% [34–41%]	38 [28–48]%	37 [28–47]%	NS
	**A/Darwin/9/2021 (H3N2)**			
% with antibodies	87% [84–89%]	93 [88–98]%	82 [74–90]%,	*p* < 0.001
GMT	121.0 [108.5–134.9]	344.8 [313.5–379.2]	49.6 [44.1–55.8]	*p* < 0.001
Protection rate	70% [67–74%]	93 [88–98]%	54 [44–64]%	*p* < 0.001
	**B/Austria/1359417/2021 (B/Victoria lineage)**			
% with antibodies	41% [37–44%]	47 [37–57]%	32 [23–41]%	*p* < 0.001
GMT	34.9 [30.9–39.4]	61.2 [49.9–75.2]	26.2 [22.9–30.0]	*p* < 0.001
Protection rate	22% [19–25%]	25 [16–33]%	20 [12–28]%	NS
	**B/Phuket/3073/2013 (B/Yamagata lineage)**			
% with antibodies	80% [77–83%]	91 [85–96]%	65 [56–74]%	*p* < 0.001
GMT	45.4 [41.7–49.5]	72.2 [62.9–82.8]	35.4 [32.0–39.2]	*p* < 0.001
Protection rate	53% [49–56%]	55 [45–64]%	51 [41–61]%	NS

## Data Availability

The data presented in this study are all included in the article. Further inquiries can be directed to the corresponding author.
